# Unusual primary HIV infection with colonic ulcer complicated by hemorrhagic shock: a case report

**DOI:** 10.1186/1752-1947-4-279

**Published:** 2010-08-20

**Authors:** Stephane Emonet, Sarah Dettwiler, Isabelle Der Hagopian, Sabine Yerly, Thomas Haustein, Susannah Strasser, Bernard Hirschel

**Affiliations:** 1 Department of Internal Medicine, University Hospitals Geneva, Rue Gabrielle-Perret-Gentil 4, Geneva, 1211, Switzerland; 2 Department of Genetic Medicine and Laboratories, University Hospitals Geneva, Rue Gabrielle-Perret-Gentil 4, Geneva, 1211, Switzerland; 3 Department of Community Medicine, University Hospitals Geneva, Rue Gabrielle-Perret-Gentil 4, Geneva, 1211, Switzerland; 4 Infection Control, Medical Directorate, University Hospitals Geneva, Rue Gabrielle-Perret-Gentil 4, Geneva, 1211, Switzerland; 5 Department of Imaging, University Hospitals Geneva, Rue Gabrielle-Perret-Gentil 4, Geneva, 1211, Switzerland

## Abstract

**Introduction:**

Timely diagnosis of primary HIV infection is important to prevent further transmission of HIV. Primary HIV infection may take place without symptoms or may be associated with fever, pharyngitis or headache. Sometimes, the clinical presentation includes aseptic meningitis or cutaneous lesions. Intestinal ulceration due to opportunistic pathogens (cytomegalovirus, Epstein-Barr virus, *Toxoplasma gondii*) has been described in patients with AIDS. However, although invasion of intestinal lymphoid tissue is a prominent feature of human and simian lentivirus infections, colonic ulceration has not been reported in acute HIV infection.

**Case description:**

A 42-year-old Caucasian man was treated with amoxicillin-clavulanate for pharyngitis. He did not improve, and a rash developed. History taking revealed a negative HIV antibody test five months previously and unprotected sex with a male partner the month before admission. Repeated tests revealed primary HIV infection with an exceptionally high HIV-1 RNA plasma concentration (3.6 × 10^7 ^copies/mL) and a low CD4 count (101 cells/mm^3^, seven percent of total lymphocytes). While being investigated, the patient had a life-threatening hematochezia. After angiographic occlusion of a branch of the ileocaecal artery and initiation of antiretroviral therapy, the patient became rapidly asymptomatic and could be discharged. Colonoscopy revealed a bleeding colonic ulcer. We were unable to identify an etiology other than HIV for this ulcer.

**Conclusion:**

This case adds to the known protean manifestation of primary HIV infection. The lack of an alternative etiology, despite extensive investigations, suggests that this ulcer was directly caused by primary HIV infection. This conclusion is supported by the well-described extensive loss of intestinal mucosal CD4^+ ^T cells associated with primary HIV infection, the extremely high HIV viral load observed in our patient, and the rapid improvement of the ulcer after initiation of highly active antiretroviral therapy. This case also adds to the debate on treatment for primary HIV infection, especially in the context of severe symptoms and an extremely high viral load.

## Introduction

Patients with primary HIV-1 infection (PHI) may have a variety of symptoms, including flulike syndrome, lymphadenopathy, gastrointestinal symptoms, pharyngitis, headache, and cutaneous lesions. The non-specific presentation and a lack of suspicion among clinicians often delay the diagnosis of PHI [1]. During this period, the patients are unaware of being highly infectious. Early diagnosis is thus essential to prevent further transmission and requires a high level of suspicion and either the use of p24 antigen or the detection of HIV RNA by polymerase chain reaction (PCR). The following case is a classic example of a missed HIV diagnosis in the context of a mononucleosis syndrome [1], with a previously undescribed complication of HIV acute infection.

## Case presentation

A 42-year-old Caucasian man of Portuguese origin, with serologic evidence of past hepatitis B infection, presented to his general practitioner with a one-week history of sore throat, fever, and fatigue. Pharyngitis was diagnosed, and amoxicillin-clavulanate prescribed. Ten days later, the patient was admitted to our hospital with swelling of his tongue and lips and a widespread itchy maculopapular rash. A grade two allergic reaction to amoxicillin-clavulanate was diagnosed, and intravenous clemastin was given, with rapid subsequent improvement of the swelling and rash. However, the patient remained febrile (38.5°C) with continuing exudative pharyngitis and painful bilateral cervical lymphadenopathy (up to three cm in diameter). Laboratory results were as follows: Hemoglobin, 136 g/L; white blood cells, 7.0 g/L with 7% non-segmented neutrophils and 15% lymphocytes; C-reactive protein, 168 mg/L; ASAT, 500 U/L; ALAT, 400 U/L; γ-GT, 1200 U/L; and total bilirubin, 36 μmol/L. A pharyngeal swab yielded mouth flora only. The patient remained hospitalized for investigation of the non-resolving mononucleosis syndrome (tonsillar pharyngitis, fever, and lymphadenopathy) with a presumptive diagnosis of viral infection complicated by drug allergy.

Review of systems only revealed loose feces for one week and was considered as a minor side effect of the antibiotic therapy.

**Figure 1:**
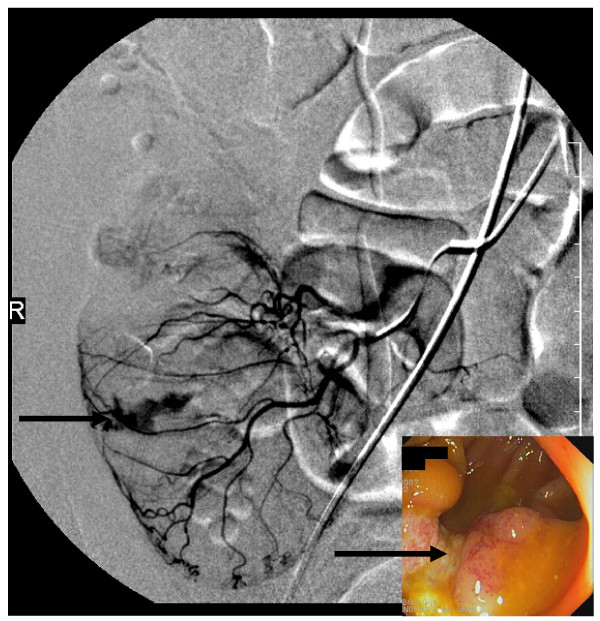
**Angiography and colonoscopy**. Caecal bleeding on angiography (arrow) and caecal ulcer on colonoscopy (arrow).

The following day, the patient collapsed with hematochezia that resulted in a decrease in his hemoglobin to 66 g/L. He was hemodynamically stabilized with intravenous saline and blood transfusions. Computed tomography showed extravasation of contrast medium into the cecum, and active bleeding was confirmed by angiography (Figure 1). After angiographic occlusion of a branch of the ileocaecal artery, bleeding stopped and did not recur. Colonoscopy revealed a large caecal ulcer with irregular margins and a fibrinous base (Figure 1), located at the site of the hemorrhage.

**Figure 2:**
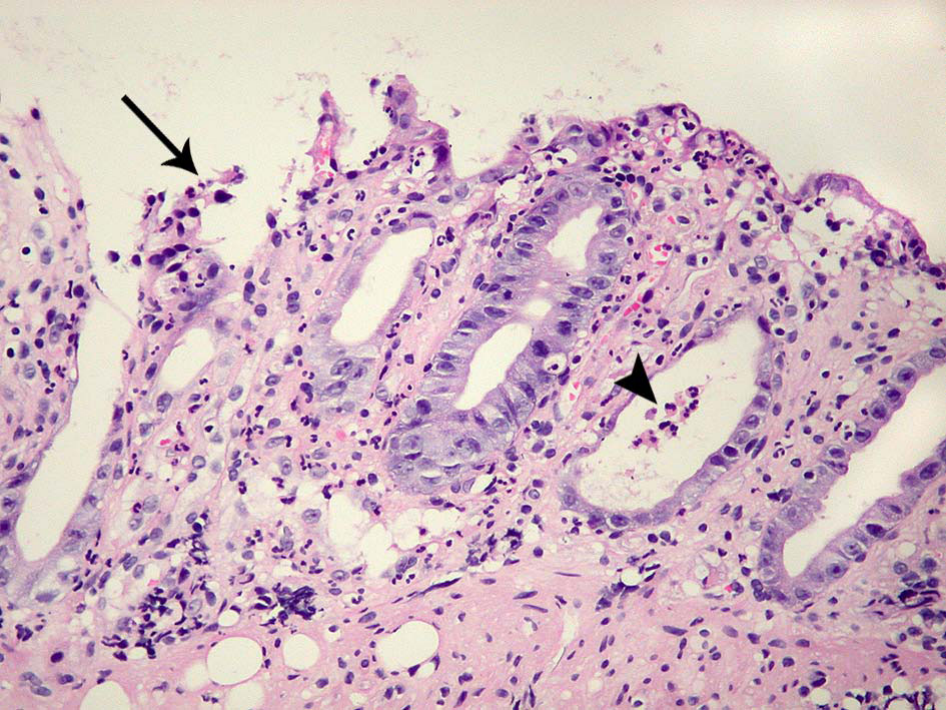
**Biopsy of the caecal ulcer**. The colic biopsy shows an acute inflammation in the lamina propria, consisting of an increase in polymorphonuclear cells. The neutrophils infiltrate the mucosa, leading to crypt abscess (arrowhead), flattened epithelial cells, and erosion (arrow).

**Figure 3:**
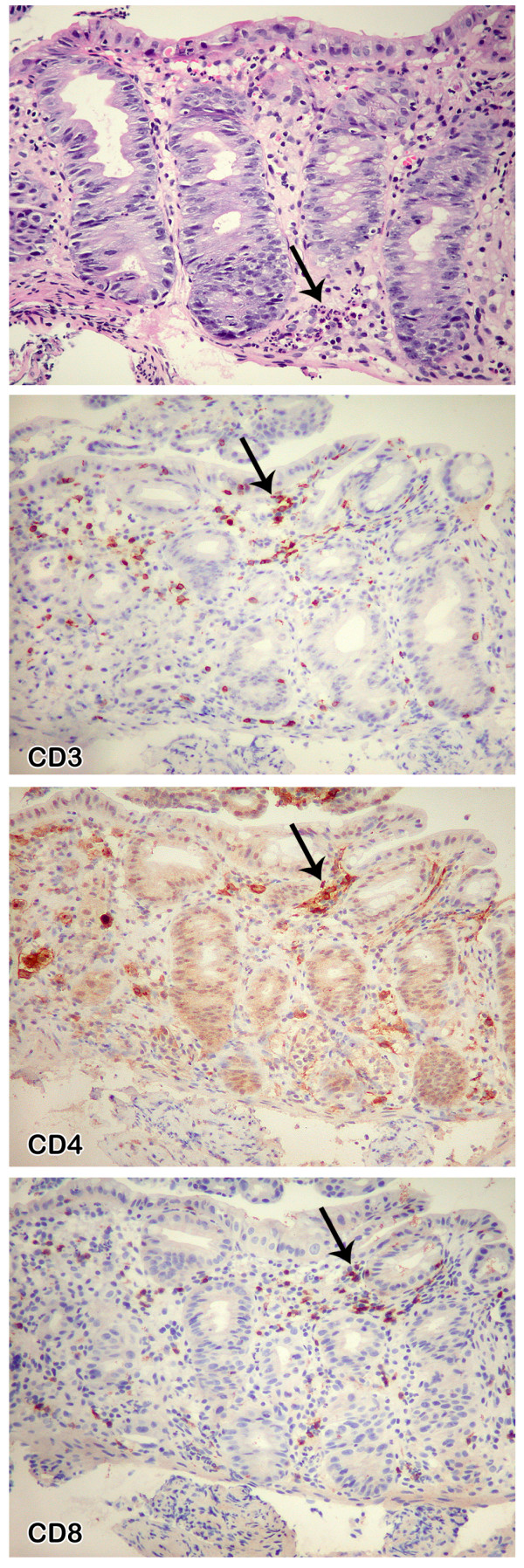
**Biopsy of colonic mucosa adjacent to the ulcer; immunohistochemistry with anti-CD3, -CD4, and -CD8**. The colic mucosa is regenerative, with less mucus in the cytoplasm of the epithelial cells. The lamina propria contains some neutrophils, which infiltrate the epithelial cells (arrow), without an increase of mononuclear cells. In this inflamed mucosa, fewer CD4^+ ^cells (arrow) than CD8^+ ^T cells (arrow) are found.

A biopsy of the ulcer showed acute inflammation in the lamina propria, consisting of an increase in polymorphonuclear cells without a concomitant increase of lymphocytes and plasma cells, associated with crypt abscesses and flattened epithelial cells (Figure 2), compatible with an edge of the ulcer. The adjacent colonic mucosa showed a mildly inflamed lamina propria with regenerative glands (Figure 3). Although lymphocytes and plasma cells were not increased in the lamina propria, immunohistochemistry with anti-CD3, -CD4 and -CD8 demonstrated fewer CD4^+ ^T cells than CD8^+ ^T cells (Figure 3). Grocott, periodic acid Schiff (PAS), Giemsa, and Ziehl-Neelsen stains, as well as immunohistochemical methods using antibodies to CMV, HSV-1, and HSV-2, did not reveal any pathogens. P24 antigen was undetectable. Tissue culture was not performed. The CMV viral load in the blood was very low (29 copies/mL).

Serologic investigations to identify a cause for the patient's mononucleosis syndrome indicated only past infections with EBV, CMV, and *T. gondii *(the presence of IgG, but not IgM antibodies). However, the patient was found to have a low CD4 count (180/mm^3^, 10% of total lymphocytes), and a fourth-generation HIV test was positive. The patient's history suggested that he had acquired his HIV infection recently. He reported having had a negative HIV rapid test (Determine) five months earlier and had had unprotected sex with a man one month previously. Primary HIV infection was confirmed by a very high viremia (3.6 × 10^7 ^copies/mL HIV-1 RNA tested by CAP/CTM, Roche) and an evolving immunoblot (INNOLIA, Innogenetics) on the blood sample obtained one day before his gastrointestinal hemorrhage.

In view of a persisting mononucleosis syndrome and a further decrease in his CD4 count (101/mm^3^, 7%), antiretroviral therapy with emtricitabine, tenofovir, and lopinavir/r was started. The pharyngitis, fever, and lymphadenopathy improved rapidly. On follow-up five months later, his viremia was less than 40 copies/mL, and his CD4 count was 310/mm^3^.

## Discussion

Oral and esophageal ulcers have been described in PHI [2,3], whereas colonic ulcerations are usually associated with AIDS [4]. However, a report exists of a lorry driver in Rwanda with melena in the context of acute HIV and oropharyngeal and rectal ulcers [5]. Esophageal ulceration and rectal fissures also were identified in a patient with HIV-1-seronegative AIDS [6]. Pancreatitis [7] and hepatitis [8] have been reported occasionally.

We believe that PHI could be the cause of this colonic ulcer for a number of reasons. First, despite extensive investigations, we could not identify any alternative infectious or tumoral etiology. Even if "special histologic stains are rarely beneficial for the evaluation of HIV-related gastrointestinal infections" [9], the failure to detect pathogens on Grocott, PAS, Giemsa, and Ziehl-Neelsen stains, as well as immunohistochemically by using antibodies to CMV, HSV-1, and HSV-2, indirectly supports the role of HIV as the causative pathogen. Second, the existing literature suggests an extensive loss of intestinal mucosal CD4^+ ^T cells associated with an increase of cytotoxic CD8^+ ^T cells during PHI [10]. Third, the exceptionally high level of HIV viremia, the temporal correlation between ulcer and primary HIV infection, and the rapid improvement of the ulcer after the initiation of highly active antiretroviral therapy, strongly suggest that HIV itself was the cause of this ulcer.

## Conclusion

This case is a reminder to always consider HIV in the differential diagnosis, especially when confronted with non-resolving symptoms or an unusual presentation. The intestinal complication described is unusual but relevant, because it links pathophysiology and clinical events.

This report also adds to the debate on treatment of acute HIV infection. Even if no documented proven advantage exists after six months of treatment [11], antiretroviral therapy (ART) is effective in alleviating the symptoms of PHI, as shown in this case. In the future, the use of CCR5 inhibitors in the context of a very recent HIV infection could become an area of special interest, because CCR5 + CD4^+ ^T cells of the gut are the primary target of HIV [12].

## Competing interests

The authors declare that they have no competing interests.

## Authors' contributions

SE and BH cared for the patient during and after his hospitalization and wrote the manuscript.

TH contributed to writing and editing of the manuscript. SD did the histologic workup of the biopsy. IDH treated the patient in the emergency center. SY determined the viral load and the immunoblot. SS performed the arterial occlusion (angiography). All authors read and approved the final manuscript.

## Consent

Written informed consent was obtained from the patient for publication of this case report and accompanying images. A copy of the written consent is available for review by the Editor-in-Chief of this journal.
